# Supplementary material for: Are carbon emissions trading and green financial instruments synergistic? -Comprehensive quantitative research based on content analysis
Source: PLoS One. 2024 Mar 7;19(3):e0298601. doi: 10.1371/journal.pone.0298601 (PMC10919601; doi:10.1371/journal.pone.0298601)
Supplement: S1 Data — (ZIP) [file pone.0298601.s001.zip › Data/地级市Z_F工作报告.zip!/╕ú╜¿/╚¬╓▌╩╨/└┤╫╘╣1⁄2╓┌║┼í╛┬φ┐╦╩2╛▌═°í┐_2013.docx]

各位代表：

　　现在，我代表泉州市人民政府向大会作工作报告，请予审议，并请市政协委员和其他列席人员提出意见。

　　一、2012年政府工作回顾

　　过去一年，在中共泉州市委领导下，市人民政府深入贯彻落实科学发展观，坚持“项目带动、转变提升、和谐进步、跨越发展”工作思路，把握机遇，迎难而上，经济社会发展取得新成效。全市实现生产总值4726.5亿元，增长12.3%；公共财政预算收入293.46亿元，增长21.2%；城镇居民人均可支配收入32283元、农民人均纯收入11915元，分别增长12.5%和12.6%，完成市十五届人大一次会议确定的目标任务。

　　过去一年，泉州发展得到上级的高度重视和大力支持。国务院批准设立泉州金融服务实体经济综合改革试验区，我市成为全国第三个金改区；国务院批准设立泉州台商投资区，实行国家级经济技术开发区政策。省政府专门出台促进泉州民营经济发展的十条措施；批准泉州开展民营经济综合配套改革试验，支持打造民营经济发展乐园。

　　2012年的主要工作和成效是：

　　1.持续砥砺前行，经济发展稳中有进。面对严峻经济形势，全力以赴保增、促稳、攻坚，稳住经济大盘。尽心尽力帮扶企业。全市召开企业座谈会226场，协调解决各类问题1672项。开展政策落实专项行动，帮助企业用好扩大生产、技术改造、自主创新等政策。注重发挥行业协会作用，召集15个重点行业协会，“一业一策”研究解决应对措施。工业支撑紧抓不放。建立促进工业稳定增长联动机制，发挥龙头企业带动作用，完成工业增加值2645.79亿元；产值超亿元工业企业增至1704家，完成增加值1941.94亿元。装备制造、建筑建材、工艺制品、食品饮料等主要行业增加值增速均超过18%。产能建设强力推进。突出生产性、生财性项目，实施县级以上产业重点项目1200个，完成年度投资1030亿元。建成海峡轨道客车、三安光电、本科电器等工业项目261个，新增年产能650亿元；实施重点技改项目290个，新增年产能750亿元；建成万达、网商创业园、英良五号、洛江中心商城等服务业项目121个，新增年销售收入550亿元。中化炼油项目获国家发改委核准。泉港、泉惠石化园区升格为省级开发区，南山片区进驻企业20家。市场开拓内外并进。成功举办海博会、鞋博会、石博会、茶博会、雕博会等泉州商品博览会系列活动。引导品牌企业优化专卖店布局，开展“电子商务在行动”，网上市场份额位居全国前列。积极参加国内外商品展，开展 “品牌泉州境外行”系列活动；全市外贸总额突破200亿美元大关，其中出口123.74亿美元，新增出口超亿美元企业6家；泉州出口加工区、晋江陆地港外贸总额分别达13.5亿美元和7亿美元。“第三产业发展年”活动成效明显。服务业重点项目完成投资413.9亿元。做好“营改增”试点工作，新增限上批零住餐单位616家、3A级以上物流企业5家，港口吞吐量突破1亿吨，机场旅客吞吐量突破200万人次。实现全社会消费品零售额1663.07亿元。开展“市长带你游”系列旅游推介活动，清源山获批国家5A级旅游景区，成功举办陶瓷文化生态旅游节；完成旅游总收入350亿元，增长22%。举办首届创意产业节，对接项目419个，意向金额20.72亿元。金融业税收增长32.9%。“五大战役”深入实施。重点项目建设、新增长区域发展、城市建设、小城镇改革发展、民生工程战役分别完成投资750亿元、1780亿元、280亿元、466.3亿元和97.2亿元，均超额完成年度投资任务。要素保障及时有力。加强资金统筹调度，市本级筹资235.68亿元，全市累计争取上级资金82亿元；市城建国投、路桥等企业退出融资平台清理目录，组建市开源实业、水利投资等公司，项目筹资渠道进一步拓宽。与5家银行开展战略合作，民生银行在泉成立石材产业金融事业部；引进菲律宾首都银行，实现外资银行零的突破；新增贷款629.89亿元，中小企业贷款余额2060亿元；新增小额贷款公司14家。电网建设超额完成年度投资任务。提升用工服务专项行动取得明显成效。获批农用地转用和土地征收5.22万亩、海域使用3万亩。

　　2.发挥实体优势，“二次创业”掀起热潮。民营经济加快转变提升。出台实施推进 “二次创业”若干意见及配套文件，启动民企转型升级扶持行动计划，设立兑现专项资金5亿元，完成规模以上民营工业增加值2043.78亿元，增长17.7%。新增上市企业3家。国家工商总局出台6条措施，支持泉州品牌国际化；新增中国驰名商标32件、马德里商标国际注册97件，泉州商标馆开馆；我市品牌闪耀伦敦奥运会，赞助22个国家体育代表团；海西国际时装周品牌效应显现。出台加快推进产业转移扶持政策，山区与沿海实现项目对接7个，总投资20.7亿元。自主创新“三年行动”深入实施。总结推广“安踏经验”，组织科技“五清零六提升”行动，与教育部合作启动“蓝火计划”。实施重点科技计划项目610个，新增高新技术企业10家、创新型企业25家、产业技术创新战略联盟9家、授权专利8208项，制修订各类标准39项，完成高新技术产业产值2025亿元。微波通信创新型产业集群、科技服务体系列入国家试点工程，入选首批国家知识产权示范城市。加快淘汰落后产能，安溪、晋江建筑饰面石材行业实现整体退出。人才支撑不断加强。出台实施服务民企“二次创业”人才计划，启动海内外泉籍人才回归工程；实行高层次人才“一站式”服务和高级人才证制度；新增院士专家工作站4个，50人（团队）被评为省级各类高层次人才，兑现386位高层次人才贡献奖励。“三维”对接成效明显。开展招商大会战，全力大招商、招大商。落地央企项目4个，总投资103亿元。开展对外大型招商活动4场，签约外资项目173个，总投资73.91亿美元，其中5亿美元以上合同项目2个，引进惠普、IBM、普利司通等世界500强企业。在珠三角民企洽谈会上签约项目110个，总投资1234亿元。举办民企“二次创业”推介会5场，出台推进异地泉籍企业回归创业12条措施，实现项目回归120个，总投资1100亿元；引进企业总部或营销中心300多家，增加税收约32亿元。

　　3.注重夯实基础，“三农”工作扎实推进。全市农林水支出39.78亿元，增长31.8%，实现农林牧渔业产值282.23亿元。6个老区县（市、区）争取上级资金补助5.2亿元。“美丽乡村”建设率先启动。实施 “六大工程”，完成593个村庄规划编制，大力推进农村垃圾处理。抓好市级新农村建设试点示范村20个、扶贫开发重点村20个、老区跨越发展工程重点帮扶村4个，评选“美丽乡村”10个。现代农业持续发展。完成粮食生产任务。新增省级以上农业龙头企业8家、农业“三品”认证产品24个，获批国家级农民专业合作社示范社16家。安溪、永春成为首批国家级出口农产品质量安全示范区。“对台合作引种创新基地”成为全国唯一支持的对台种业项目。争取上级渔业补助7.7亿元，获批设立湖洋溪国家级水产自然保护区和2家远洋捕捞企业。水利建设全面推进。76个水利项目完成投资35.1亿元。启动白濑水利枢纽工程前期工作，彭村、八峰、双溪水库和晋江防洪工程试验段等重大水利项目开工。“两江”上下游水资源保护补偿资金提高到每年2亿元，重点流域综合整治完成投资17.18亿元，晋江流域省控断面Ⅲ类水质达标率100%，13个集中式饮用水源地水质达标率100%，38万农村人口饮水安全问题得到解决。崇武至秀涂海岸带资源环境保护整治完成投资8075万元。桃溪流域综合治理模式在全省推广。率先在全国建成水资源动态管理系统。防灾减灾工作持续加强。农村生产生活条件不断改善。完成农村电网改造升级260公里、公路安全保障625公里、危（病）桥改造27座，完成 “造福工程”搬迁10500人、危旧房改造7000户。建设省级农村环境连片整治示范区5个、村庄环境整治试点村17个。完成植树造林35.24万亩、水土流失治理39.47万亩，补充耕地1.44万亩，建设高标准基本农田4万亩。

　　4.凝聚环湾合力，城市建设加快拓展。深化“城市建设管理年”活动，363个项目完成投资365亿元。交通网络日臻完善。晋石高速、金安高速、田安大桥、黄龙大桥和台商区南北主干道等建成通车，泉州湾跨海大桥、南石高速、莆永高速、国道324晋鲤段、东海大街拓改等加快推进，实现县县通高速的历史性突破。泉州港通过世界卫生组织口岸核心能力达标考核。泉州晋江机场改建主体工程完工，航空口岸通过国家对外开放验收。环湾片区加快建设。中心城区建成区面积176.5平方公里。编制完成市绿道系统、桃花山生态区保护建设等规划。晋江、石狮、南安城市发展向“两江一湾”靠拢，晋江两岸城市建设水平不断提升。东海、城东、北峰丰州、晋江滨江、江南等环湾新区初步形成，台商区完成固定资产投资88亿元。滨海公园、桃花山公园一期、金山公园慢线等基本完工，城东学园、一院新院二期等加快推进，东海学园、江南学园、中医联合医院等开工建设。出台实施中心市区“三旧”改造政策，城市更新步伐加快。城市环境不断优化。实施绿化、美化、亮化、净化提升工程，完成10条街路景观整治和沈海高速森林生态景观通道建设，中心市区新增绿地面积530万平方米。48个“点线面”试点项目完成投资14.82亿元。完成田安南路、安吉路等供水管网改造，市区水质不断改善。全市新投入运营污水处理厂15座，城东垃圾转运站等一批环卫设施投入使用。完成电力线路缆化改造26公里。城市管理持续加强。推行市政公用行业常态化考评，组建市容市貌观察团，抓好14条主干道精细化管理。社区建设和物业管理水平持续提升。地理信息公共服务平台、数字城市管理平台开通，成为“全国数字城市建设示范市”。开展违法建房整治活动。投入公共交通9000万元，取消市区公交空调收费，公交服务水平进一步提高。

　　5.突出惠民利民，社会保持和谐稳定。社会保障体系更加健全。新增城镇就业15万人、农村劳动力转移就业6万人，培训劳动者25.6万人次。参加职工“五险”人数增加43万人次，新农合、城镇居民医保参保率分别达99.5%和95%。城乡居民养老保险参保率达97%，新增乡镇敬老院16所、社区居家养老服务站150个、农村老年体育活动中心22个，泉州老年公寓、江南老年颐乐园顺利推进，成立老龄服务志愿者协会。完善城乡最低生活保障制度，发放低保金2.75亿元。建成泉州市福乐家园和2所县级社会福利中心。全市慈善组织累计筹集善款33.7亿元，投入慈善救助活动20.7亿元；侨捐公益事业超过5亿元。物价总水平保持稳定。教育事业优先发展。4个区（市）完成“教育强县”创建或通过省级预评，7个区（市）通过义务教育基本均衡发展评估。新增义务教育标准化学校290所、公办幼儿园70所。配备国标校车130部，完成校安工程重建任务。晋江获批异地高考试点。安溪茶学院正式招生。建成青少年社会教育活动中心20个。医改工作扎实推进。新增医院床位2109张。石狮公立医院改革试点经验在全省推广，全市所有基层医疗卫生机构和1485家村卫生所实施药品零差率销售。改善农村医疗卫生条件“三百工程”、基本公共卫生服务项目全面完成。文化蓬勃发展。完成文化产业增加值215亿元。闽南文化生态保护区“十百千基础工程”基本完成。木偶戏传承人培养计划入选联合国“非遗”优秀实践名册，泉州木偶剧院开工建设。“侨批档案”入选“亚太地区记忆遗产”，“海丝”、“闽南红砖建筑”入选中国“世遗”预备名单。梨园戏剧团、南音乐团摘获国家文艺类最高奖。国家级、省级工艺美术大师分别新增3位和40位。发行德化陶瓷特种邮票。完成14家国有文艺院团改革，组建泉州广播电视台。公共图书馆、文化馆免费对外开放，实现农家书屋行政村全覆盖。举办泉州市第31届戏剧会演、“闹元宵·闽南文化周”、美国“泉州电视周”、土耳其“泉州文化周”，以及海峡两岸商会经济论坛、闽台佛教文化交流周等海峡论坛泉州系列活动。不断加强体育工作，拓展南少林武术国际交流，推动五祖拳进校园；我市运动员在伦敦残奥会上取得“两金一铜”好成绩。深化文明城市创建工作，城市文明程度指数居全国提名资格城市前列。保障性安居工程任务超额完成。新开工4万套，完成投资90.23亿元；基本建成2.89万套，新增配租配售1.61万套。新增归集住房公积金36亿元、发放贷款27亿元。国家环保模范城市通过复核。生态市创建走在全省前列，完成节能减排任务，市区空气质量优良率达98.5%。社会管理持续创新。全面推行流动人口居住证制度，成为全国农民工最具幸福感城市、全国首批流动人口社会融合示范工程试点城市。推进综合警务改革创新，“保平安创满意促和谐”行动成效明显。加强领导干部接访群众工作，做好矛盾纠纷排解防范。推进安全生产标准化、执法检查常态化建设，开展道路交通安全综合整治“三年行动”和集中整治大会战，安全生产事故4项指数全面下降。食品药品安全工作扎实推进。全市人口出生率13.45％。，政策符合率93.36%，保持低生育水平。蝉联全国双拥模范城“七连冠”。其他工作有效推进。国防动员、民兵、预备役、海防、人防、支前、审计、统计、方志、档案、气象、防震减灾、离退休干部服务等工作取得新成绩。

　　6.力促效能提速，政府建设得到加强。自觉接受人大及其常委会监督，依法执行人大决定决议，主动支持人大常委会开展执法检查、人大代表视察及专项调研。支持政协履行职能，主动接受政协民主监督，积极采纳委员调研成果。办理人大代表建议406件、政协委员提案516件，满意和基本满意率均为100%。完成农业、教育、科技法定投入，实现第三个五年依法治市规划目标。国有资产监管进一步加强。建立财政专项资金管理、投融资项目资金管理、国有资本经营预算等制度。推行“马上就办”，建立民企直通直达服务制度，实现审批改革提速“两个50%”目标。加强机构编制管理，完成事业单位清理规范工作。深化反腐倡廉。启动“服务型政府”教育培训工程。完成市委、市政府29项为民办实事项目。

　　各位代表，回顾攻坚克难、负重奋进的2012年，我们经受住严峻经济形势的考验，各方面工作取得积极进展，成绩来之不易。这是省委、省政府和市委坚强领导的结果，是市人大、市政协和社会各界监督支持、共同努力的结果，是全市广大人民群众团结奋斗的结果。在此，我代表市人民政府，向全市广大干部群众，人大代表、政协委员，各民主党派、工商联、无党派人士、各人民团体、离退休老同志，驻泉省部属单位、驻泉部队、武警官兵、企业界人士和来泉创业务工人员，以及广大港澳台同胞、海外侨胞、海内外朋友，表示衷心的感谢和崇高的敬意！

　　同时，我市发展仍然存在一些困难和问题，主要是：产业转型升级还不够快，发展后劲、创新活力有待增强；不少企业生产经营面临资金短缺、库存增加、成本上升等多重压力；市本级财力十分有限，重点项目融资困难；中心城市辐射带动能力还不够强，现代文明程度有待提高，“三农”工作存在薄弱环节；节能减排、淘汰落后产能任务繁重，生态环境保护有待加强；民生改善、社会管理与人民群众期望仍有差距；一些公务人员工作作风和服务水平有待提升。2012年度指标中，公共财政总收入和第三产业增加值未完成预期目标。对此，我们要进一步增强忧患意识和责任意识，采取有力措施，认真加以解决。

　　二、2013年政府工作的指导思想、目标任务、主要措施

　　当前，我市发展正处于十分难得的战略机遇期。党的十八大胜利召开，全国上下掀起全面建成小康社会热潮，海西战略深入推进，泉州金融改革、民营经济改革获批实施，全市人民倍感振奋，创业激情日益高涨，泉州发展空间更加广阔。同时，必须清醒看到，世界经济低速增长态势仍将延续，我国经济平稳运行压力不小。我们一定要凝聚力量，攻坚克难，开拓创新，以更大作为、更优业绩、更新成效，赢得主动、赢得优势、赢得未来。

　　2013年是我市实施“十二五”规划、提前实现全面建成小康社会的关键一年。政府工作指导思想是：全面贯彻落实党的十八大精神，以邓小平理论、“三个代表”重要思想、科学发展观为指导，认真落实“五位一体”的总体布局，围绕市委“两个加快”，以提高经济增长质量和效益为中心，推进产业、港口、城市联动发展，突出集群发展、创新驱动、环湾建设、改革开放、民生优先，打造金融服务实体经济综合改革试验区和民营经济综合配套改革试验区，在更高起点上建设经济强市和美好家园。

　　2013年主要预期目标为：地区生产总值增长11.5%；公共财政总收入增长13.5%，公共财政预算收入增长13.5%；工业增加值增长13.5%；全社会固定资产投资增长22%；外贸出口增长8%；实际利用外资（历史可比口径）增长6%；社会消费品零售额增长15.5%；居民消费价格总水平涨幅控制在3.5%左右；城镇居民人均可支配收入增长11%；农民人均纯收入增长11%；城镇登记失业率控制在2%以内；人口自然增长率控制在8.2％。以内；完成省下达的节能减排任务。为此，我们要按照市委决策部署，稳中求进，好中求快，力求更快，确保完成上述目标任务。

　　1.推进产业发展，致力在创新转型、优化结构上求突破

　　加快产业结构战略性调整，大势所趋，刻不容缓。要深化 “二次创业”，坚定不移推动转变提升，加快形成新的经济发展方式，打造产业发展新优势，进一步增强经济实力。

　　壮大提升产业集群。深化产业升级“四化”工程、“百千万亿”倍增计划，加快纺织服装、鞋业、石化向3000亿元产业集群迈进，装备制造、建筑建材向千亿元产业集群迈进，形成一批超百亿元、500亿元的产业集群。坚持高端发展，实施质量强市战略，推进“两化”融合示范建设，支持企业开展资本运作、管理创新、品牌建设，培育设计师品牌，组建大师创作团队，发展大师经济，进一步提升传统产业。坚持龙头带动，实施恒安智能化生产、佳龙石化二期等192个重点工业项目，总投资2381亿元，确保中化炼油、联合石化“脱瓶颈”、百宏化纤、玖龙纸业等项目投产，培育壮大成长型产业和战略性新兴产业。坚持产业集聚，建好4个国家级、10个省级开发区以及各类专业园区，推进产业项目向园区集中，加快形成规模生产力和效益。坚持整合优化，开展“万家企业手拉手专项行动”，突出行业内部资源整合、产能对接、平台共享、联合销售，支持龙头企业与中小微企业开展协作配套，推动沿海产业向山区转移，提高产业集群整体竞争力。同时，认真落实帮扶企业各项政策和制度，重点扶持遇到困难的产业链关键项目、龙头企业和成长型企业。

　　做大做强现代服务业。深化“第三产业发展年”活动，组织实施项目382个，年度投资506.6亿元。大力发展现代物流业，实施海峡箱包物流商城、观音山现代物流园区、为为国际城等项目，新增3A级以上物流企业5家，开展外挂车船回迁专项行动。培育壮大金融、工业设计、文化创意、服务外包等产业。围绕打造旅游强市，加强戴云山脉旅游区域联盟合作，办好茶文化旅游节，抓好德化石牛山、永春北溪、安溪温泉世界等建设，策划引进文化、滨海、生态旅游大项目，把旅游业培育成为战略性支柱产业。

　　大力发展海洋经济。启动新一轮市级海洋功能区划修编，培育临海产业带、滨海城镇带和蓝色景观带。做大做强临港工业，建设石狮海洋生物产业园、南安海峡科技生态城等项目，培育壮大华宝等一批海洋生物医药企业。大力发展港口经济，争取开通更多国内外航线。壮大现代渔业，推进海产品深加工，支持发展远洋捕捞业。抓好石井、围头湾区域用海项目。推进海域、海岸、海岛整治开发和生态修复。挖掘弘扬海洋文化。

　　强化创新驱动发展。全市安排财政科技投入7.6亿元，全社会研发投入增长30%以上。做好自主创新“三年行动”考核验收，开展科技支撑产业升级“双百工程”，实施重点技改项目200个以上、科技计划项目650个，培育一批“科技小巨人企业”。加强泉州高新区“一区八园”建设，抓好数据灾备中心、联通云计算中心等项目，培育园区主导产业。强化知识产权创造和运用，力争每万人发明专利拥有量1.4件以上。推进标准制修订工作。实施“科技创新服务平台全面提升计划”，密切产学研合作，鼓励民营资本投入创建新型研发机构，建设中科院海西研究院泉州装备制造研究所、微波通信研究院、物联网研究院、石狮甲骨文纺织服装创新发展云平台及一批国家级质检中心、检测实验室。

　　突出人才优先发展。落实完善人才政策。加强各类高层次人才的培养、引进和使用工作。强化人才公共服务，鼓励民间资本参与人才市场、人才创业园等建设，培育引进人力资源服务机构，解决人才落户、居住、子女教育等问题。引导企业建设博士后工作站、院士专家工作站，与院校合作开展“订单式”人才培养工作。建好“海内外泉籍优秀人才信息库”，鼓励青年企业家创业，促进大中专毕业生就业。

　　2.增进人民福祉，致力在保障民生、改善民生上求突破

　　从维护广大人民根本利益的高度，多谋民生之利，多解民生之忧，加快健全基本公共服务体系，努力让人民过上更好生活。

　　进一步办好人民满意的教育。新改扩建公办幼儿园77所。加快城东、东海、江南学园建设，启动台商区国际教育园区项目；推进城镇中小学扩容，新增学位1万个；实施农村义务教育薄弱校改造工程68个，新增标准化学校110所。保障来泉务工人员子女平等接受教育。统一城乡义务教育生均公用经费标准。加强职业教育实训基地建设。支持办好华侨大学、泉州师院等高校。加快学校体育发展，增强中小学生体质。强化校园、校车安全管理。引导社会力量办学，扶持老年教育、特殊教育和社区教育。做好关心下一代工作。

　　进一步完善社会保障体系。推动更高质量就业，完善职业培训补贴、紧缺工种免费培训制度，新增城镇就业15万人。加强城镇职工“五险”和城镇居民医保、新农合工作，健全城乡居民大病保险体系。大力发展老龄服务事业和产业，完善城乡居民养老保险制度，建设提升150个社区居家养老服务站，拓展老龄志愿服务，关怀帮助“空巢老人”和“失独家庭”。健全社会保障经办管理体制。加快“金保工程”建设。加强城乡低保、医疗救助和灾害救助，提高农村五保供养水平，抓好市社会福利中心建设，大力发展慈善事业。保障妇女儿童合法权益。

　　进一步提高人民健康水平。深化医药卫生体制改革，出台落实进一步方便群众就医的政策措施。合理布局、积极发展优质医疗资源，抓好儿童医院拓改、中医联合医院、医大附属二院东海分院等建设，引导社会力量办医，新增医院床位2300张。推进县级公立医院改革试点，扶持乡镇卫生院发展，实现村卫生所药品销售零差率全覆盖。抓好卫生人才队伍建设。强化重点传染病、慢性病防控。加强精神卫生工作。坚持计划生育基本国策，深化“生育文明·幸福家庭”促进计划，综合治理出生人口性别比失衡问题，充分发挥计生协会作用，促进人口长期均衡发展。大力发展体育事业，支持创建全省全民健身先进县（市、区）工作。

　　进一步为群众做好事办实事。抓好市区水质优化、特殊群体健康关爱、贫困残疾儿童康复救助、特困失能老人救助、农村低保家庭安居房、青少年社会教育活动中心、乡村学校少年宫、农村老年体育活动中心、副食品基地建设、消防能力提升等30项为民办实事项目，进一步解决好群众关心的居住、饮水、出行、养老、教育、健康、卫生、救助、物价、安全等问题。

　　3.突出扩大内需，致力在投资带动、消费拉动上求突破

　　牢牢把握扩大内需这个战略基点，发挥投资对经济增长的关键作用，培育消费增长点，不断增强长期发展后劲。

　　深入实施“五大战役”。进一步拓展战役内涵，扩大战役规模，确保每个战役取得更大成效。实施重点项目460个，年度投资778亿元，确保开工90个、竣工100个。强化战役保障，深化政银企合作，增加并引导好民间投资；开展国土资源节约集约模范县（市）创建活动，提高土地利用效率；创新安征迁工作机制。

　　突出重大基础设施建设。力争南石、莆永高速公路竣工通车，加快泉州湾跨海大桥、沈海复线、安溪连接线等高速项目建设，做好厦沙高速等前期工作；建成中化青兰山配套码头，建设石湖、肖厝、斗尾、秀涂、小岞等一批深水泊位及泉州湾深水航道二期工程；抓好福厦铁路惠安站、湄洲湾南岸支线、中化专用线等铁路项目，做好吉永泉铁路和城市轨道交通前期工作；持续实施泉州晋江机场改建项目；抓好鸿山热电厂二期等能源项目。

　　不断扩大消费需求。科学布局都市商圈、特色商业街和便民商业网点，深化“万村千乡市场工程”和“新农村现代流通经营服务网络工程”，建设一批大型超市、农贸市场。提升家政、文化娱乐、健身休闲等服务消费，发展网络购物、社区商业服务。加强食品药品监管，治理“餐桌污染”，做好“菜篮子”基地食品安全示范工作，扩大肉品质量安全信息可追溯试点，深化药品安全示范县创建。稳定物价总水平，正确引导社会消费预期。

　　4.强化“三农”基础，致力在统筹发展、城乡一体上求突破

　　按照“保供增收惠民生、改革创新添活力”要求，加大统筹城乡发展力度，大力推进“美丽乡村”建设，让广大农民平等参与现代化进程、平等享受现代化成果。

　　全面建设“美丽乡村”。认真实施五年行动计划及“六大工程”，按照“四美”要求，抓好一批“美丽乡村”示范村，带动一批新农村示范村、扶贫重点村、老区帮扶村和环境整治示范村建设。编制完成270个村庄规划，强化农村住房建设管理。加快农村石头房、危旧房改造，实施“造福工程”。深化家园清洁行动。广泛开展文明乡村创建活动。

　　加快发展现代农业。增强农业综合生产能力、防灾减灾能力和可持续发展能力。加强粮食生产、粮食安全和农产品有效供给；深化"131"强龙工程，壮大高效特色农业，扶持发展现代茶业、花卉产业和林下经济，培育休闲农业、创意农业示范点。强化农业科技支撑，新增农业“三品”认证产品20个。抓好重大动植物疫病防控，加强农产品质量安全监管。构建新型农业经营体系，发展新型经营主体、新型农民合作组织和多元服务主体，培育市级农民专业合作示范社40家，推广应用“世纪之村”农村信息化综合服务平台。

　　完善农村基础设施。实施80个水利项目，加快晋江防洪工程、彭村水库以及市区应急备用水源等建设步伐，抓好白濑水利枢纽、惠女水库引调水、山美水库生态保护试点等项目，加强农村饮水安全、中小河流治理、农田水利等工作。持续推进农村公路提级改造、危（病）桥改造、安全保障工程和电网改造升级。

　　健全“三农”支撑体系。进一步加大“三农”投入，认真落实强农惠农富农政策。采取综合措施，不断开辟农民就业门路，拓宽增收渠道。壮大村集体经济实力。深化农村综合改革，探索农村集体建设用地流转，提高农民在土地增值收益中的分配比例，深化集体林权制度及其配套改革。推动安溪、南安、永春、德化申报中央苏区县。扶持“老少边岛”地区加快发展。

　　5.构筑环湾城市，致力在同城发展、提升品位上求突破

　　城镇化是未来发展的最大潜力。要以环湾的意识统揽全局，坚持以同城化的标准建设，统筹推进中心城市、县域、小城镇发展，倾力打造“环湾活力都市、滨海幸福之城”。

　　塑造组团式空间格局。坚持“统一规划、拼图作业”，完善提升环湾城市规划体系，继续开展“城市建设管理年”活动，确保完成投资340亿元。加快东海、城东、北峰丰州、江南池店、洛江新城、台商区、晋江滨江、蚶江祥芝、霞美、崇武山霞等环湾重大片区开发进度，做到优先布局项目、优先安排资金、优先保障用地。提升清濛园区城市化水平。建设南北翼新城。

　　增强现代化城市功能。提升环湾城市交通体系，完善向湾通道、湾区主干道，抓好普贤路拓改、国道324丰洛段改造、台商区东西主干道拓改、崇武至秀涂滨海大道改造及旅游开发、石狮环湾大道等建设，实施刺桐路北拓、北峰至城东通道、金屿大桥、百崎大桥等项目。培育环湾商务区、金融区、会展区，加快东海、鲤城、晋江滨江总部经济区以及恒安、移动、电力等总部大楼建设步伐，力争泉州会展中心、东海五星级酒店、北峰潘山启动区开工。完善公共服务设施，抓好城市规划建设及成就展示馆、工人文化宫及大会堂建设；统筹建好各类市政工程，规划建设一批停车场，推进交通节点改造；做好城市地质调查工作，加强城市地下空间综合利用。

　　打造人性化宜居环境。突出城市绿能建设，启动江滨北路等8条市政道路景观改造提升，全面推进“点线面”整治，抓好北环城河带状公园绿道、桃花山至滨海公园绿道和泉州植物园建设，建成中心市区慢线系统网络，创建国家生态园林城市。打造一批优质宜居小区，加快安置房、城市综合体、“三旧”改造建设步伐；确保完成省下达的保障性住房建设任务，发挥公积金的住房保障作用，保持房地产健康发展；加强社区建设，提升物业管理水平。实施中心市区积水内涝整治工程。强化城市管理综合考评，突出市容市貌、户外广告等整治，实现市区主干道精细化管理全覆盖，确保通过国家卫生城市复评。坚持公交优先发展，优化环湾公交系统。发挥“数字城管”平台作用。建设“智慧泉州”。

　　提高城镇化发展质量。按照“一湾两翼多支点”城镇布局，编制完善城镇化发展规划，推动经济社会发展规划、城市总体规划、土地利用总体规划“三规融合”，引导城镇化健康发展，确保城镇化率达61.5%。实施“大城关”战略，大力发展县域经济，支持石狮全域城市化综合配套改革，抓好惠安西部新城和安溪、永春、德化县城建设。深化小城镇改革发展，发挥21个试点镇的示范带动效应，支持有条件的乡镇建立综合执法、行政审批等平台。优化城镇产业布局，加快产城融合发展。重视解决农业转移人口市民化问题，统筹安排户籍、土地、财税、住房、教育、医疗、社会保障等体制、政策、利益调整，推动基本公共服务从户籍人口向常住人口覆盖、从城市社区向小城镇和农村延伸。

　　6.深化改革开放，致力在体制创新、拓展空间上求突破

　　改革是发展的最大动力、最大红利。要以更大的勇气和智慧，深化经济体制改革，全面提高开放型经济发展水平。

　　深化重点领域改革。全面落实金融服务实体经济综合改革试验区总体方案，围绕打造海西“实体金融服务中心”，重点突破引进银行机构、发展股权交易市场、设立民间资本管理公司和民间借贷登记服务中心、改制上市、企业直接债务融资、支持小微企业发展、建设区域征信系统和诚信体系等工作，拓宽金融资本和产业资本的通道，促进实体经济发展壮大。贯彻落实民营经济综合配套改革试验的若干意见，致力在提升产业、自主创新、集聚发展、制度创新、民间投资、对外开放、土地保障等方面先行先试。支持行业协会在市场开拓、行业规划、协作配套等发挥更大作用。加强市级财源建设，增强市级财政统筹发展能力和调控能力。做大做强国有企业，健全国资监管体制。提升“三维”对接成效。持续开展“大招商、招大商”，重点引进大型央企、知名民企、世界500强企业。深度挖掘在泉央企潜力。深化“回归工程”，发挥异地商会作用，引进更多泉商参与“二次创业”；开展与全国民企100强和行业龙头企业项目对接活动。创新外商投资促进举措。健全招商项目快速落地机制，提高履约率、开工率、投产率。推进泉州开发区深度开发和官桥园区建设。

　　开拓国际国内市场。转变外贸发展方式，发挥出口加工区、陆地港和4个省级出口基地的作用；巩固传统市场，拓展自贸区市场，扩大自主品牌、高新技术产品出口，鼓励引进先进技术和设备；持续开展“品牌泉州境外行”活动，支持企业收购国际品牌；深化大通关建设。抢占扩大国内市场，凸显泉州商品博览会效应，发挥专卖店渠道优势，加快海峡化工产品交易中心、国际轻纺城、南安光机电贸展中心等建设；大力发展电子商务，出台实施电子商务发展三年规划，创建全国电子商务示范城市和示范基地。

　　深化与台港澳侨合作。全面实施台商投资区总体规划，加快基础设施、产业园区、社会事业建设，力争在引进重大台资项目上实现突破。积极对接台湾电子、光电、机械、石化等产业，促进台资企业转型升级；加快台湾农民创业园、台湾农业技术新品种交流推广中心建设；办好海峡论坛泉州活动、第六届海峡两岸 “农订会”，拓展对台客运、货运、小额贸易三条通道，密切对台交流合作和人员往来。发挥泉港泉澳泉台经济合作促进委员会作用，落实与港澳《关于进一步深化合作的框架协议》。加强外事侨务工作，扩大友城交流，举办第二届世界泉州同乡恳亲大会。

　　7.打造文化强市，致力在引领风尚、弘扬传承上求突破

　　文化是人民的精神家园。要坚持先进文化前进方向，发挥泉州传统文化优势，增强文化自觉和文化自信，提高文化软实力。

　　深化文明城市创建工作。开展社会主义核心价值体系学习教育，不断提高市民文明素质，努力形成知荣辱、讲正气、守诚信、作奉献、促和谐的良好风尚。注重人文关怀和心理疏导，加强未成年人思想道德建设和大学生思想政治教育。广泛开展志愿服务。抓好政务诚信、商务诚信和社会诚信建设。

　　推进公共文化繁荣发展。深化文化体制改革，鼓励文化艺术创作创新，推动传统文化与现代元素融合，创作优秀作品，造就名家大师和文化领军人物。规划建设地标性文化街区，抓好泉州木偶剧院、市图书馆新馆、泉州歌舞剧院等项目。发挥文艺院团在促进文化繁荣中的重要作用。大力实施文化惠民服务工程，完善四级公共文化设施服务网络。加快第二轮修志步伐。

　　挖掘深厚文化底蕴优势。加强历史文化名城、闽南文化生态保护核心区建设，办好2013世界闽南文化节，建立世界闽南文化展示中心，健全优秀文化传承体系、文化遗产保护体系、文化资源开发体系。推进“海丝”、“闽南红砖建筑”和“侨批档案”申遗。鼓励申报省级、国家级、世界级“非遗”项目。积极抢救保护濒危传统工艺美术品种技艺。

　　进一步壮大文化产业。重点发展十大文化产业，争创国家广告产业试点园区，实施泉州当代艺术馆、领SHOW天地、源和1916、市工艺美术馆、晋江“三创园”、石狮服饰创意园区等项目，培育一批文化骨干企业。推进文化与科技、信息、旅游深度融合，发展新型文化业态，做足环清源山、泉州古城、崇武古城等文化旅游文章，把文化优势转化为文化生产力。培育壮大文化市场。建好晋江、南安、石狮体育产业基地。

　　8.建设生态文明，致力在环境保护、绿色发展上求突破

　　树立尊重自然、顺应自然、保护自然理念，加快形成节约资源和保护环境的产业结构、生产方式、生活方式，建设美丽泉州。

　　推动资源利用方式根本转变。严格节能减排奖惩措施，大力发展循环经济，实施100个重点节能技改项目，加强对134家企业的节能管理，做好德化陶瓷产业园区循环化改造示范试点工作。开展工业落后产能普查，完善落后产能退出机制。推进印染行业转型升级，抓好印染集控区建设。强化排污许可证制度，推进造纸、皮革、电镀、建陶、石材等重点行业深度治理，加强污水处理厂及其配套管网的建设管理。打造节水型社会。

　　突出环境整治。强化水、大气、土壤、噪音等污染防治，启动细颗粒物（PM2.5）监测，推进机动车尾气排放、工程建设扬尘等专项治理。实施100个重点流域综合整治项目，建立崇武至秀涂海岸带资源环境保护长效机制。突出工业园区环境集中治理，深化农村环境连片整治，完成规模化畜禽养殖场全过程治理。

　　加强生态建设。力争通过省级生态市验收。抓好北渠节水改造、泉州湾河口湿地等生态工程，综合整治主要水源地、水库、生态脆弱地区以及废弃矿井、采坑，治理水土流失40万亩、“青山挂白”49.28万平方米，增强生态产品生产能力。完善环境风险防范和应急管理制度。深化“四绿”工程建设，创建省级森林县城2个，完成造林绿化29.3万亩，加强森林防火。

　　9.构建和谐社会，致力在管理创新、保障安全上求突破

　　社会和谐人人有责，和谐社会人人共享。要提升社会管理科学化水平。完善流动人口居住证制度，创新流动人口和特殊人群服务管理；健全重大决策社会稳定风险评估机制；完善大调解工作体系，推广“警民联调”，建立市县乡村四级工作平台；推行城乡社区“网格化”管理，创建和谐社区。要深化平安建设。构建县乡村三级巡逻防控体系，深化专项打击整治行动，提高群众安全感和满意率；创建“安全发展城市”，加强重点行业、重点领域专项整治，完成企业安全生产标准化达标任务；深化交通安全综合整治“三年行动”，加大道路交通安全隐患排查治理；完善突发公共事件应急处置机制。要增强促进和谐的合力。启动第四个五年依法治市规划，深化“六五”普法，建设法治泉州；做好国防动员、支前、征兵、双拥、优抚安置和人民防空工作，巩固发展军政军民团结；开展民族团结进步创建活动，依法管理宗教事务；支持工、青、妇等人民团体发挥作用，推动社会组织健康有序发展。

　　三、打造高效率、负责任、服务型的政府

　　全面建成小康社会已经进入“倒计时”，人民对美好生活的向往，考验着政府的履职能力。我们一定要牢记使命，脚踏实地奋斗，扎扎实实工作，用实干共同托起“中国梦”。

　　1.勇于拼搏、勇于开拓。面对加快转型、加快发展的双重任务、双重压力，我们要更加解放思想、奋发有为，以更高的站位、更大的气势谋划推动发展，能快则快、能超则超，保持全省平均水平之上；要更加爱拼敢赢、大胆先行，抓住倒逼出的新机遇，建好两个试验区，用改革激发潜力，用改革释放红利，实现更高质量的发展；要更加凝心聚力、海纳百川，发挥民办特区、侨台资源、开放前沿的独特优势，汇聚推动泉州发展的正能量。

　　2.求真务实、埋头苦干。要突出“马上就办”，拿出精气神，提高执行力，做只争朝夕、真抓实干的行动者。要大力发扬“滴水穿石、人一我十”的精神，不图虚名、不务虚功，把心思用在干事业上，把力量用在抓落实中。要模范遵守“八项规定”，调研须扎实，活动要踏实，会议求实效，文件讲实际。要深化简政放权，抓好国家级行政服务标准化试点，营造“认不认识一个样”、“熟不熟悉一个样”的办事环境。

　　3.根植人民、造福人民。干部怎么干，决定群众怎么看。我们要始终与人民心心相印、与人民同甘共苦、与人民团结奋斗，夙夜在公，勤勉工作。要加快政府职能转变，把更多注意力聚焦到社会管理上，把更多财力用在改善民生上，把更多精力放在服务群众上，让人民感受变迁、触摸幸福、实现理想。要积极开展接访、走访、回访活动，真诚倾听群众呼声，真实反映群众愿望，真情关心群众疾苦。

　　4.依法行政、清正廉洁。要自觉接受人大法律监督、工作监督和政协民主监督，认真办理人大议案、代表建议和政协委员提案，广泛接受民主党派、社会公众、新闻舆论监督。健全行政执法、行政复议和规范性文件审查备案制度。深化政务公开和办事公开。推进事业单位分类改革。贯彻落实机关事务管理条例，强化公共节能。健全惩防体系，推进廉政风险防控，加强效能督查、绩效管理，着力整治庸懒散奢，以良好形象取信于民。

　　各位代表，让我们在中共泉州市委领导下，紧紧依靠和团结全市人民，励精图治，锐意进取，全力推进现代化泉州建设，不愧今天的责任和使命，不负明天的梦想和追求！
